# Development and validation of outcome prediction models for acute kidney injury patients undergoing continuous renal replacement therapy

**DOI:** 10.3389/fmed.2022.853989

**Published:** 2022-08-18

**Authors:** Bo Li, Yan Huo, Kun Zhang, Limin Chang, Haohua Zhang, Xinrui Wang, Leying Li, Zhenjie Hu

**Affiliations:** Intensive Care Unit, Hebei Medical University Fourth Affiliated Hospital and Hebei Provincial Tumor Hospital, Shijiazhuang, China

**Keywords:** acute kidney injury, continuous renal replacement therapy, prediction model, nomogram, validation

## Abstract

**Object:**

This study aimed to develop and validate a set of practical predictive tools that reliably estimate the 28-day prognosis of acute kidney injury patients undergoing continuous renal replacement therapy.

**Methods:**

The clinical data of acute kidney injury patients undergoing continuous renal replacement therapy were extracted from the Medical Information Mart for Intensive Care IV database with structured query language and used as the development cohort. An all-subset regression was used for the model screening. Predictive models were constructed *via* a logistic regression, and external validation of the models was performed using independent external data.

**Results:**

Clinical prediction models were developed with clinical data from 1,148 patients and validated with data from 121 patients. The predictive model based on seven predictors (age, vasopressor use, red cell volume distribution width, lactate, white blood cell count, platelet count, and phosphate) exhibited good predictive performance, as indicated by a C-index of 0.812 in the development cohort, 0.811 in the internal validation cohort and 0.768 in the external validation cohort.

**Conclusions:**

The model reliably predicted the 28-day prognosis of acute kidney injury patients undergoing continuous renal replacement therapy. The predictive items are readily available, and the web-based prognostic calculator (https://libo220284.shinyapps.io/DynNomapp/) can be used as an adjunctive tool to support the management of patients.

## 1. Introduction

Acute kidney injury (AKI) is a critical comorbidity and a global health problem with high morbidity and high mortality (1–3). In the intensive care unit (ICU), the morbidity can be as high as 50% (4). Since there are no specific drugs for AKI, renal replacement therapy (RRT) plays a major role in treatment (2). Although there is currently no evidence that continuous RRT (CRRT) is superior to intermittent RRT (IRRT) (5–7), CRRT is often preferred for hemodynamically unstable patients (2). However, among these patients, even with appropriate CRRT, there is still very high mortality (8), and the cost of treatment is often high. Thus, it is important to develop reliable tools that can inform expectations regarding outcomes and decisions regarding treatment.

Clinical predictive models can estimate the probability of a patient's outcome through the statistical implementation of a series of clinical characteristics of the patient, and may be helpful for patient management as a decision support tool (9). Currently, the most widely used outcome prediction models in the ICU are the Acute Physiology and Chronic Health Evaluation II (APACHE II) classification system (10) and the Sepsis-related Organ Failure Assessment (SOFA) score (11). However, these models do not focus on outcome prediction in AKI patients undergoing CRRT. Several prediction models have been published (12, 13), but there are some limitations in clinical practice, such as improper variable selection strategies, difficulty of use in clinical settings and a lack of generalizability to different settings. Therefore, there is an urgent need develop an easy-to-use predictive tool that supports clinical decision-making.

We developed and validated outcome prediction models of AKI patients treated with CRRT.

## 2. Methods

### 2.1. Data source

The development cohort included 1148 patients who were recruited from Medical Information Mart for Intensive Care IV (MIMIC IV version 1.0) (14, 15). MIMIC IV is a relational database containing the real information of patients admitted to the ICUs of Beth Israel Deaconess Medical Center in Boston, MA, USA, from 2008 to 2019. The principal investigator completed the Human Research Course (Record ID: 37097306) and obtained access to this database, and the project was approved by the institutional review boards of the Computational Physiology Laboratory of the Massachusetts Institute of Technology and Beth Israel Deaconess Medical Center and was granted a waiver of informed consent. All data were extracted with structured query language (SQL) from BigQuery.

The validation cohort included 121 patients treated in the Department of Intensive Care Unit, Fourth Hospital of Hebei Medical University, Shijiazhuang, China. This study was approved by the Ethics Committee of the Fourth Hospital of Hebei Medical University (approval number: 2021KS034).

### 2.2. Patient involvement

The inclusion criteria in this study were as follows: (1) AKI patients meeting the KDIGO-AKI criteria; and (2) patients who received CRRT after diagnosis. Patients younger than 18 years were excluded, and when the same patient were admitted multiple times, only data for the first admission was included. In addition, in the validation cohort, patients whose family members voluntarily stopped treatment within 24 h after receiving CRRT were also excluded.

### 2.3. Diagnosis and outcomes

AKI was defined as any of the following Kidney Disease Improving Global Outcomes (KDIGO) criteria (16): increase in SCr ≥0.3*mg*/*dl* (≥26.5*mol*/*l*) within 48 h; increase in SCr ≥1.5 times baseline, which is known or presumed to have occurred within the prior 7 days; or a urine volume < 0.5*ml*/*kg*/*h* for 6 h.

The primary outcome was defined as death within 28 days after receiving CRRT. Patients in the validation cohort whose family members voluntarily stopped treatment for more than 24 h were considered dead.

### 2.4. Variable extraction

The following variables were extracted from the relevant literature and clinical records:

**Demographic characteristics:** Age (17–21), sex (20, 21), height, and weight (21).

**Comorbidities:** Congestive heart failure (CHF) (18), atrial fibrillation (AF), chronic liver disease (CLD), chronic obstructive pulmonary disease (COPD), chronic coronary syndrome (CCS) (18), hypertension, diabetes, and malignant cancer (18, 19).

**Last vital signs within 2 h prior to receiving CRRT:** Heart rate (HR) (18), mean arterial pressure (MAP) (18, 21), and temperature (T).

**Results of the last laboratory test within 24 h prior to receiving CRRT:** White blood cell count (WBC), hemoglobin (HB) (17, 20), red cell volume distribution width (RDW) (22), platelet count (PLT) (18, 20, 21), sodium (20), potassium (20), calcium, phosphate (18, 23, 24), total bilirubin (TBIL) (18, 20, 21), albumin (18, 20, 21), creatinine (18, 21), baseline creatinine (20, 21), pH (17, 20), oxygenation index (21), base excess (20), and lactate (20, 21). Oxygenation index is calculated by equation *PaO*_2_/*FiO*_2_.

**Interventions 24 h prior to receiving CRRT:** Mechanical ventilation (18, 20, 21), vasopressor use (20, 21), sedative use, and analgesic use.

Central venous pressure (CVP) (missing rate: 74.7%), mean platelet volume (25) (missing rate: 100%), troponin (missing rate: 73.9%), N-terminal pro B type natriuretic peptide (NT-proBNP) (missing rate: 97.4%), and creatine kinase (missing rate: 70.2%) were not extracted due to excessive amounts of missing data (missing rate >50%), and there appears to be no evidence of their relationship with prognosis in this group of patients.

### 2.5. Handling of missing data

In the development cohort, there were missing data for most variables. Variables with excessive amounts of missing data were excluded. We assumed that the data were missing at random and filled in missing data using multiple imputation with chained equations. We performed fifty multiple imputations and merged the dataset into the development dataset. All analyses were performed with R software (version 4.1.1; R Foundation for Statistical Computing).

### 2.6. Model development

We used a Q-Q plot to assess the normality of the continuous variables, and cubic spline functions were used to assess the linearity of the relationship. Continuous variables that did not conform to normal or linear distributions were converted to categorical covariates based on their clinical significance. The continuous variables are expressed as the mean (standard deviation), and the categorical covariates are reported as numbers and percentages.

All variables were included in the logistic regression model, and we added an interaction term between mechanical ventilation and oxygenation index. The variables were screened using an all-subset regression, with the best model judged by adjusting the r-squared and Bayesian information criterion (BIC). The screened models were tested for multicollinearity by calculating the variance inflation factor (VIF).

Finally, we used the best model to construct a nomogram that could provide clinicians with an intuitive and quantitative tool for predicting the outcomes of AKI patients undergoing CRRT.

### 2.7. Model validation

The model discrimination was evaluated with the C-index and area under the receiver operator characteristic curve (AUC). The model calibration was evaluated with Brier scores and calibration plots. Decision curve analysis (DCA) curves were used to assess the clinical applicability of the model (26, 27).

Internal validation was performed with the enhanced bootstrap technique, in which regression models were fitted in 1,000 bootstrap replicates, drawn with replacement from the development cohort. The model was refitted in each bootstrap replicate and tested using the original sample to estimate optimism in the model performance. External validation was performed with the validation cohort.

## 3. Results

### 3.1. Model development

**Figure 1:**
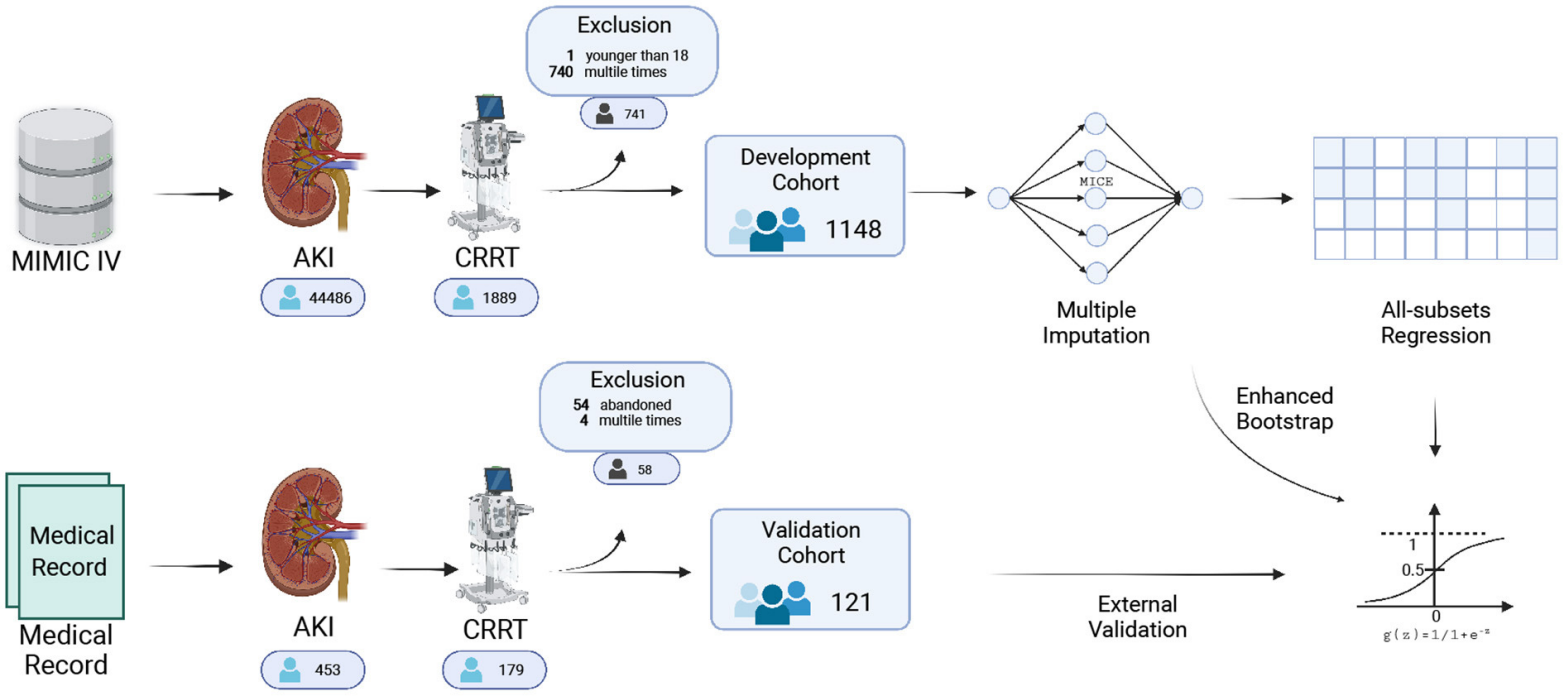
Flow chart of this study.

**Figure 2:**
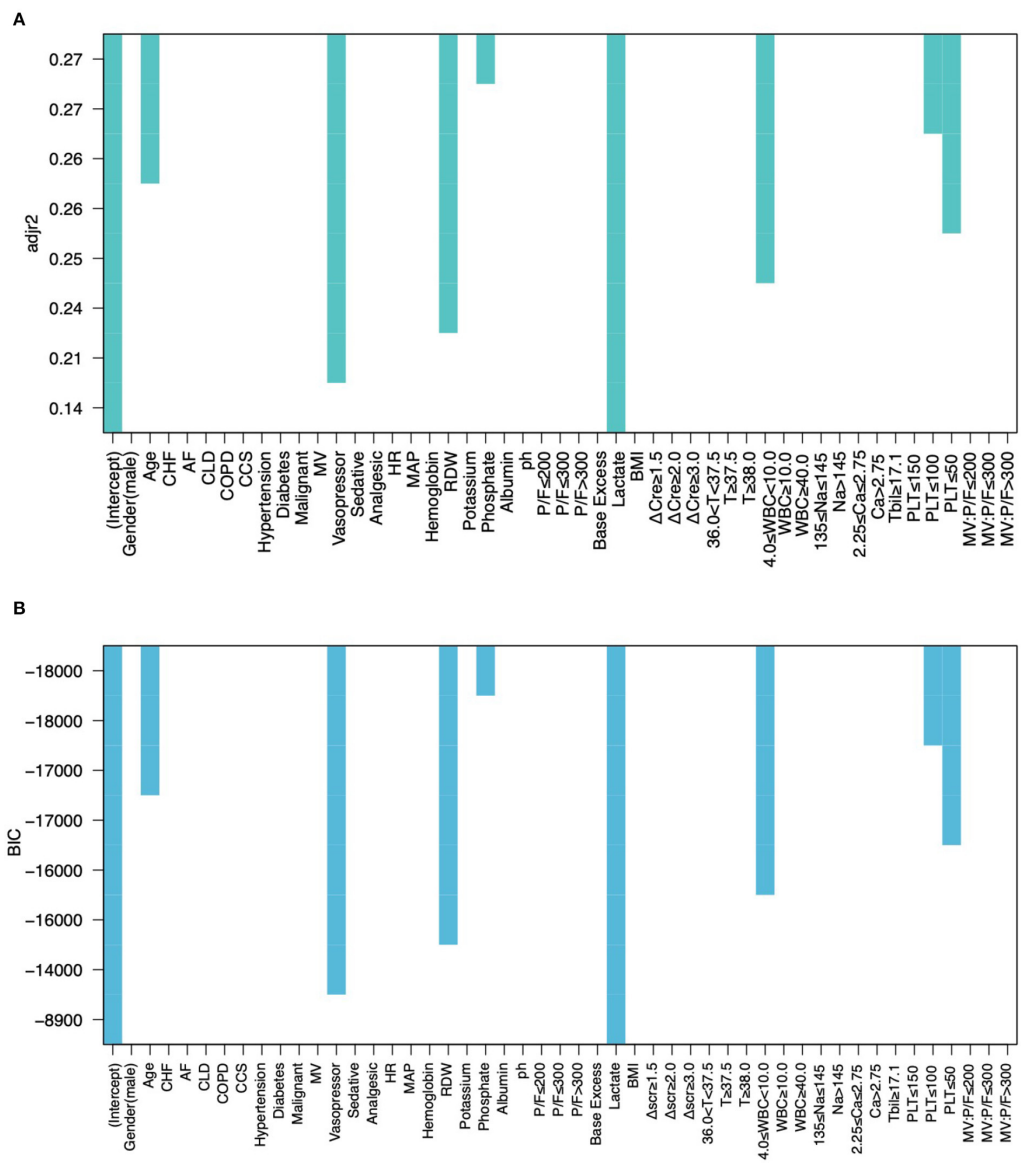
All-subsets regression by adjusting the r-squared value **(A)** and BIC **(B)**.

In total 1,148 patients from the MIMIC IV database were eventually included in our study (Figure 1). The 50 datasets obtained by multiple imputation techniques were merged into the final development cohort (Table 1). The best models were screened by adjusting the r-squared value and BIC (Figure 2).

**Table 1:** Clinical characteristics of the development cohort.

	**Overall**	**Survival**	**Death**
*N*	1,148*50	662*50	486*50
Sex (male) [*n*(%)]	34,800 (60.6)	19,950 (60.3)	14,850 (61.1)
Age [mean (SD)]	63.17 (14.61)	62.60 (14.68)	63.95 (14.48)
BMI [mean (SD)]	31.37 (8.28)	31.02 (8.06)	31.84 (8.56)
CHF [*n* (%)]	12,150 (21.2)	7,150 (21.6)	5,000 (20.6)
AF [*n* (%)]	16,200 (28.2)	9,850 (29.8)	6,350 (26.1)
CLD [*n* (%)]	9,350 (16.3)	4,550 (13.7)	4,800 (19.8)
COPD [*n* (%)]	7,600 (13.2)	4,300 (13.0)	3,300 (13.6)
CCS [*n* (%)]	16,200 (28.2)	9,900 (29.9)	6,300 (25.9)
Hypertension [*n* (%)]	6350 (11.1)	3,100 (9.4)	3,250 (13.4)
Diabetes [*n* (%)]	23,500 (40.9)	14,950 (45.2)	8,550 (35.2)
Malignant cancer [*n* (%)]	7,650 (13.3)	3600 (10.9)	4,050 (16.7)
MAP (*mmHg*) [mean (SD)]	73.71 (13.79)	75.83 (14.13)	70.83 (12.76)
HR (*bpm*) [mean (SD)]	88.66 (19.44)	85.79 (19.17)	92.56 (19.11)
Temperature (°C) [*n* (%)]			
<36.0	7,210 (12.6)	3515 (10.6)	3695 (15.2)
[36.0, 37.5]	41,331 (72.0)	24,776 (74.9)	16,555 (68.1)
[37.5, 38.0]	4,330 (7.5)	2,368 (7.2)	1,962 (8.1)
≥38.0	4,529 (7.9)	2441 (7.4)	2,088 (8.6)
WBC (*10^9^/*L*) [n(%)]			
<4.0	2,535 (4.4)	1,120 (3.4)	1,415 (5.8)
[4.0, 10.0]	17,198 (30.0)	12,220 (36.9)	4,978 (20.5)
[10.0, 40.0]	36,139 (63.0)	19,188 (58.0)	16,951 (69.8)
≥40.0	1528 (2.7)	572 (1.7)	956 (3.9)
Hemoglobin (*g*/*dL*) [mean (SD)]	9.29 (1.75)	9.31 (1.72)	9.26 (1.78)
RDW (%) [mean (SD)]	17.05 (2.67)	16.67 (2.34)	17.57 (2.99)
PLT (*10^9^/*L*) [n(%)]			
>150	24,543 (42.8)	16,170 (48.9)	8,373 (34.5)
≤ 150	11,659 (20.3)	7,170 (21.7)	4,489 (18.5)
≤ 100	14,989 (26.1)	7,372 (22.3)	7,617 (31.3)
≤ 50	6209 (10.8)	2,388 (7.2)	3821 (15.7)
Sodium (*mmol*/*L*) [n(%)]			
<135.0	19,410 (33.8)	11,644 (35.2)	7,766 (32.0)
[135.0,145.0]	33,517 (58.4)	20,001 (60.4)	13,516 (55.6)
>145.0	4473 (7.8)	1455 (4.4)	3,018 (12.4)
Potassium (*mmol*/*L*) [mean (SD)]	4.77 (0.97)	4.70 (0.94)	4.87 (1.00)
Calcium (*mmol*/*L*) [n(%)]			
<2.25	45,339 (79.0)	26,116 (78.9)	19,223 (79.1)
[2.25, 2.75]	11,499 (20.0)	6,674 (20.2)	4,825 (19.9)
>2.75	562 (1.0)	310 (0.9)	252 (1.0)
Phosphate (*mmol*/*L*) [mean (SD)]	2.08 (0.79)	1.95 (0.75)	2.26 (0.82)
Total bilirubin ≥ 17.1 μ*mol*/*L* [n(%)]	36,292 (63.2)	19,085 (57.7)	17,207 (70.8)
Albumin (*g*/*dL*) [mean(SD)]	2.91 (0.73)	2.98 (0.71)	2.82 (0.74)
Creatinine/Baseline creatinine [n(%)]			
<1.5	4,578 (8.0)	3,363 (10.2)	1,215 (5.0)
≥1.5	5,527 (9.6)	3,653 (11.0)	1,874 (7.7)
≥2.0	12,982 (22.6)	7,150 (21.6)	5,832 (24.0)
≥3.0	34,313 (59.8)	18,934 (57.2)	15,379 (63.3)
pH [mean (SD)]	7.31 (0.11)	7.34 (0.10)	7.28 (0.12)
Oxygenation index [*n* (%)]			
≤ 100	6,409 (11.2)	3,086 (9.3)	3323 (13.7)
[100, 200]	22,440 (39.1)	12,171 (36.8)	10,269 (42.3)
[200, 300]	18,407 (32.1)	11,345 (34.3)	7,062 (29.1)
>300	10,144 (17.7)	6498 (19.6)	3646 (15.0)
Base excess (*mmol*/*L*) [mean (SD)]	−5.48 (6.38)	−3.89 (5.67)	−7.64 (6.65)
Lactate (*mmol*/*L*) [mean (SD)]	3.93 (4.27)	2.54 (2.61)	5.83 (5.25)
Mechanical ventilation use [*n* (%)]	18,250 (31.8)	9,500 (28.7)	8,750 (36.0)
Vasopressor use [*n* (%)]	34,900 (60.8)	15,200 (45.9)	19,700 (81.1)
Sedative use [*n* (%)]	38,800 (67.6)	20,600 (62.2)	18,200 (74.9)
Analgesic use [*n* (%)]	42,900 (74.7)	22,700 (68.6)	20,200 (83.1)

^*^BMI, body mass index; CHF, congestive heart failure; AF, atrial fibrillation; CLD, chronic liver disease; COPD, chronic obstructive pulmonary disease; CCS, chronic coronary syndromes; MAP, mean arterial pressure; HR, heart rate; WBC, white blood cells count; RDW, red cell volume distribution width; PLT, platelet count.

**Figure 3:**
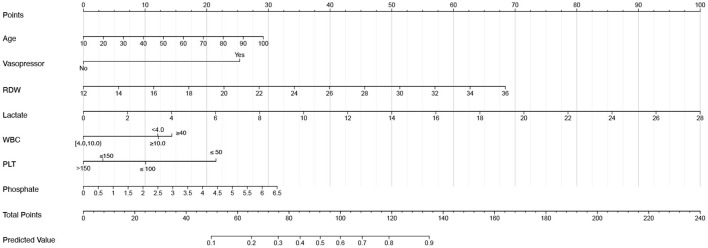
The nomogram for acute kidney injury patients undergoing continuous renal replacement therapy.

**Figure 4:**
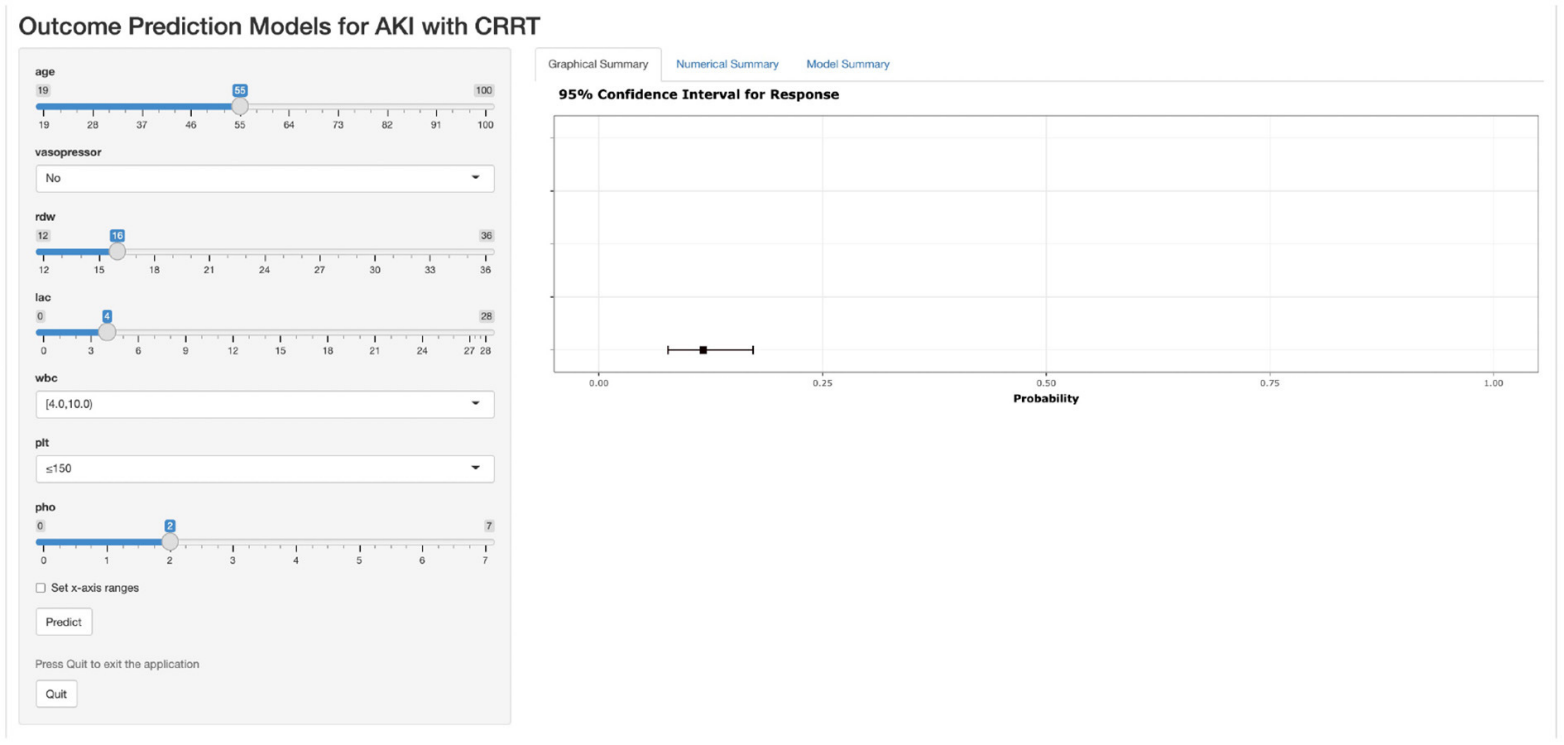
The web-based prognostic calculator.

The VIFs of the screened variables were all <5. Seven variables (age, vasopressor use, RDW, lactate, WBC, PLT, and phosphate) were finally included in our model, which was used to plot the nomogram (Figure 3) and make the web-based prognostic calculator (Figure 4, https://libo220284.shinyapps.io/DynNomapp/).

**Figure 5:**
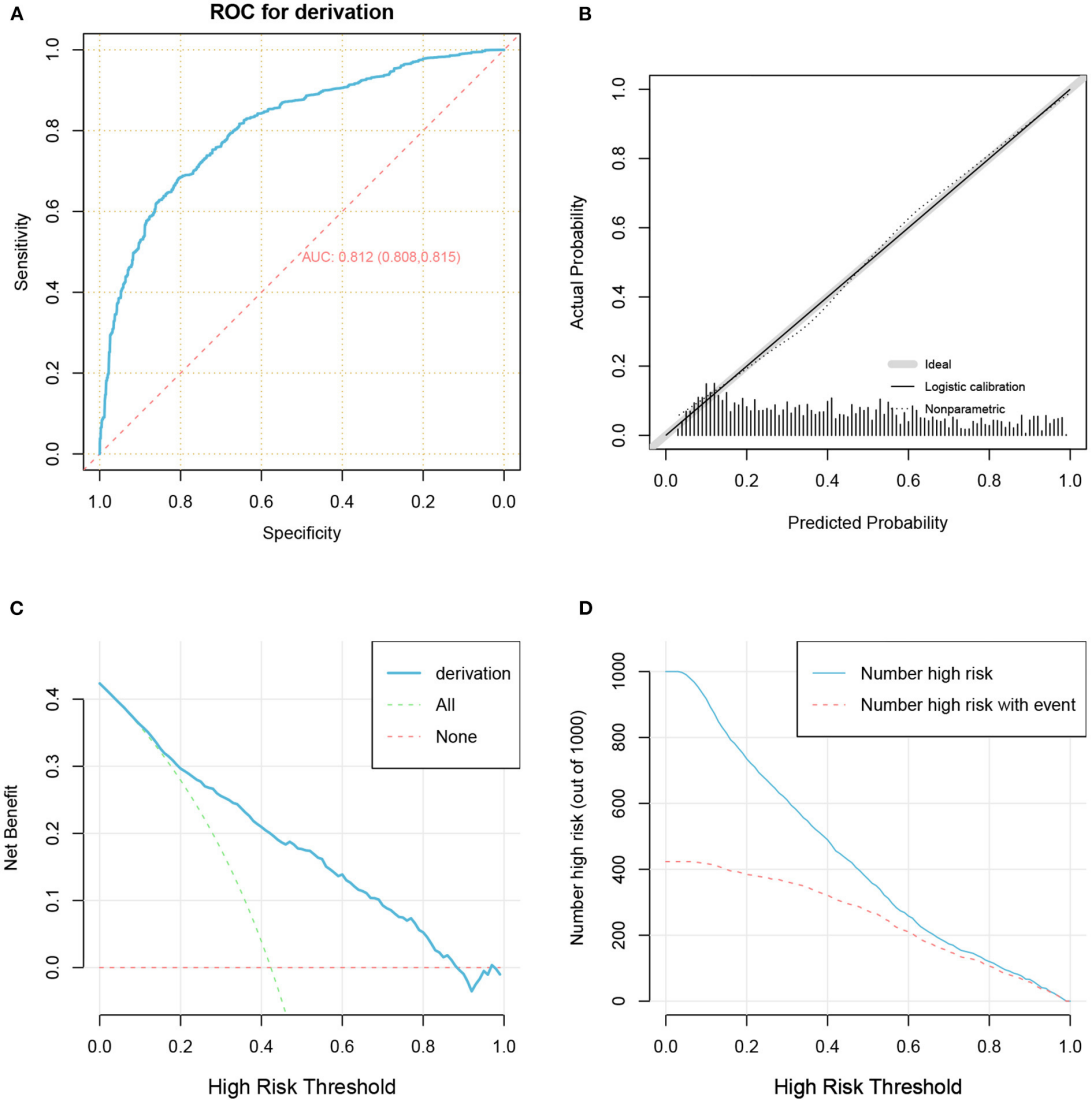
The receiver operator characteristic curve **(A)**, calibration plots **(B)**, decision curve analysis curves **(C)**, and clinical impact curve **(D)** for model in the development cohort.

The predictive performance of our model as measured by the C-index was 0.812 (Table 2 and Figure 5A) in the development cohort, indicating that the model had relatively good discriminative capacity. Our model showed high agreement between the actual and predicted probabilities in the development cohort, with a Brier score of 0.173 (Table 2 and Figure 5B). In addition, the DCA curve demonstrated that our model was clinically useful in the development cohort (Figures 5C,D).

**Table 2:** The performance in model development, internal validation, and external validation.

	**C-index**	**Brier score**
Development	0.812	0.173
Internal validation	0.811	0.173
External validation	0.768	0.202

### 3.2. Internal validation

Our model also achieved good internal validation performance after 1,000 bootstrap replicates, with a C-index of 0.811 and a Brier score of 0.173 (Table 2).

### 3.3. External validation

**Figure 6:**
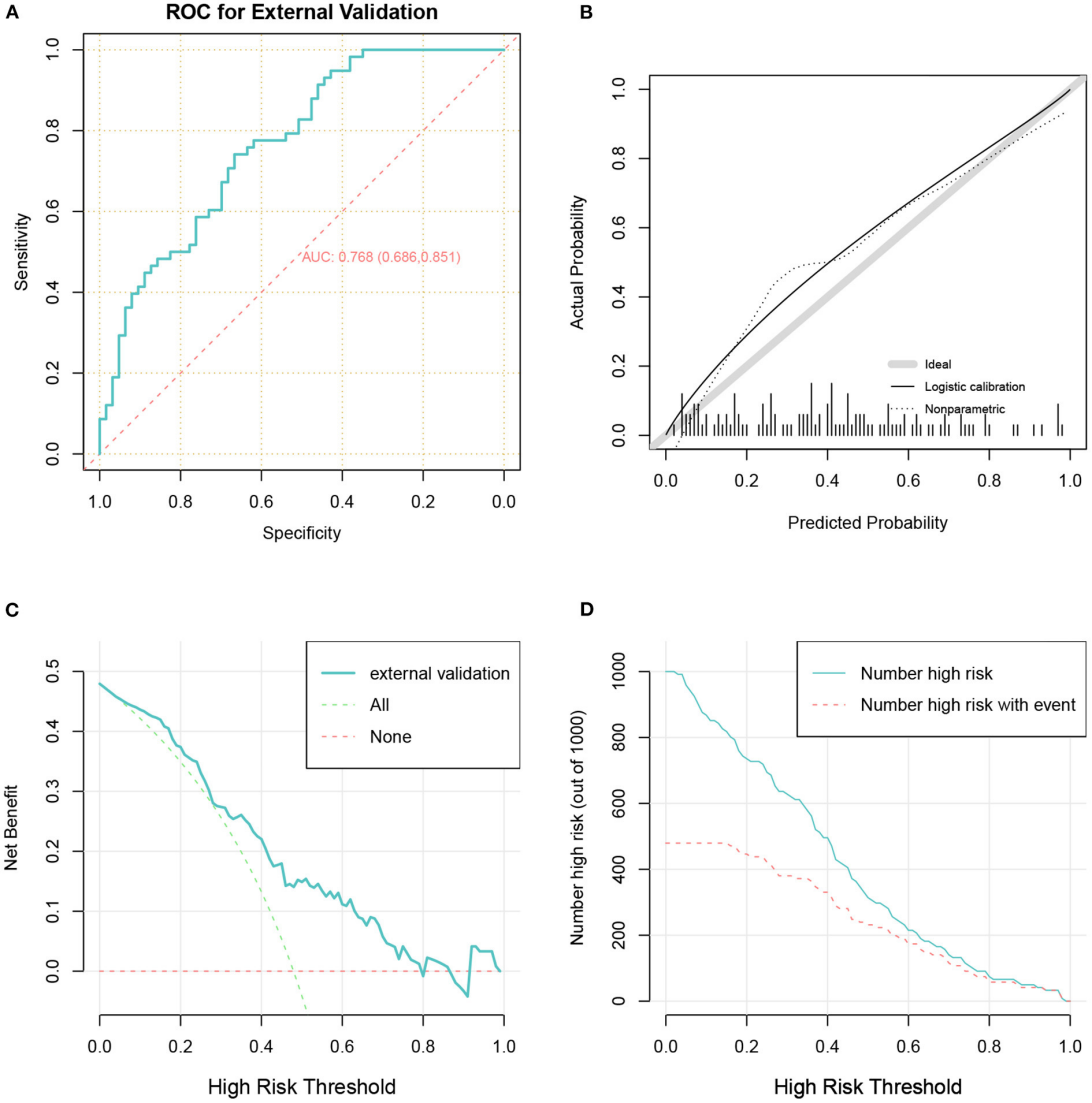
The receiver operator characteristic curve **(A)**, calibration plots **(B)**, decision curve analysis curves **(C)**, and clinical impact curve **(D)** for model in the validation cohort.

In total 121 patients were eventually included in the external validation cohort (Table 3 and Figure 1). The predictive performance of the nomogram as measured by the C-index was 0.768 (Table 2 and Figure 6A) in the external validation cohort, indicating that the model had relatively good discriminative capacity and generalizability in different settings. The nomogram also showed acceptable agreement between the actual and predicted probabilities in the external validation cohort, with a Brier score of 0.202 (Table 2 and Figure 6B). In addition, the DCA curve demonstrated that our model was clinically useful in different settings (Figures 6C,D).

**Table 3:** The predictor items of external validation cohort.

	**Overall**	**Survival**	**Death**
*N*	121	63	58
Age [mean (SD)]	62.56 (14.79)	57.32 (14.18)	68.26 (13.36)
Vasopressor use [*n* (%)]	88 (72.7)	36 (57.1)	52 (89.7)
RDW (%) [median [IQR]]	14.90 [13.90, 16.20]	14.50 [13.70, 15.35]	15.60 [14.60, 17.12]
Lactate (*mmol*/*L*) [mean (SD)]	3.80 (3.83)	2.89 (2.43)	4.79 (4.74)
WBC (*10^9^/*L*) [*n* (%)]			
<4.0	4 (3.3)	2 (3.2)	2 (3.4)
[4.0, 10.0]	28 (23.1)	17 (27.0)	11 (19.0)
[10.0, 40.0]	87 (71.9)	42 (66.7)	45 (77.6)
≥40.0	2 (1.7)	2 (3.2)	0 (0.0)
PLT (*10^9^/*L*) [*n* (%)]			
>150	53 (43.8)	30 (47.6)	23 (39.7)
≤ 150	24 (19.8)	11 (17.5)	13 (22.4)
≤ 100	23 (19.0)	13 (20.6)	10 (17.2)
≤ 50	21 (17.4)	9 (14.3)	12 (20.7)
Phosphate (*mmol*/*L*) [mean (SD)]	1.69 (0.70)	1.59 (0.68)	1.81 (0.71)

^*^RDW, red cell volume distribution width; WBC, white blood cells count; PLT, platelet count.

## 4. Discussion

AKI is common in the ICU, and although a subset of small studies has shown that preventive measures, and the rapid identification of AKI can lead to improved outcomes (28–30), patients entering the ICU often already have AKI, thus in clinical practice in the ICU, ICU physicians tend to focus more on the treatment and prognosis of AKI than on the prevention and diagnosis of AKI.

CRRT plays an important role in the management of AKI in the ICU. Since not all patients with AKI ultimately benefit from CRRT, patients, their relatives and clinicians need reliable information regarding prognosis such that they can effectively participate in shared decision-making. This is important because they are unlikely to rely solely on clinician experience and intuition when making treatment decisions.

With the widespread use of electronic medical record systems in clinical settings, “big data” and clinical medicine are becoming inseparable. From the perspectives of volume, speed, and diversity, the ICU is a wonderful combination of “big data” and clinical medicine (31). In such an era of big data, the organic combination of medical informatics and big data analytics provides a fertile new ground for analyzing the management of AKI (32, 33). Prediction tools provide an opportunity to improve AKI management in the era of big data.

Numerous predictive models of acute kidney injury are available (34), but few models are available for patients with AKI who are receiving CRRT (18, 35–37). Therefore, we aimed to obtain a reliable tool to predict the 28-day mortality in this group of patients. It is essential to clarify that although the use of Major Adverse Kidney Events (MAKE) has been suggested as a composite endpoint for such studies (38). Such a composite endpoint was also used in the SEA-MAKE score developed by Sukmark et al. (39). Twenty-eight day mortality was chosen as the single endpoint in this study. The primary considerations are as follows: First, the significant advantage of the composite endpoints is that it increases the number of events, but in patients with AKI undergoing CRRT, mortality would have been high enough and a better solution might have been to use a multivariate outcome with different outcomes, but due to the limitations of the study, this issue needs to be considered in future studies. Second, we did not know which predictors contributed to each component of the composite outcomes. Finally, even with the current definition of MAKE, death is still the most serious and important outcome of a concern. Therefore, mortality was ultimately chosen as the outcome variable in this study.

Ultimately, the prediction models performed robustly in a validation cohort from different geographical regions, time periods, and settings of care. The predictors in our model are readily available, and the nomogram and web-based prognostic calculator could facilitate clinical adoption.

### 4.1. Comparison with previous studies

Several prediction models of the outcome of AKI patients with CRRT have been developed, although their clinical use is rare.

Kim et al. (12) developed the MOSAIC model for patients with AKI undergoing CRRT. Unfortunately, this model only incorporated APACHE II outcomes and SOFA scores, and although these data were extremely accessible, they did not consider several other indicators that have predictive value and are readily available. A study by Oh et al. (22) showed that RDW was an independent predictor of the 28-day mortality in patients with AKI receiving CRRT. Phosphate reflected disease severity and predicted mortality in AKI patients undergoing CRRT in the studies by Jung et al. (23, 24). Both RDW and phosphate were included in our study. In addition, we considered additional comorbidities and laboratory indicators.

Machine learning algorithms have also been applied to predict outcomes in AKI patients undergoing CRRT (13). Machine learning algorithms appear to provide better predictive performance than traditional models, but their hard-to-interpret nature may also lead to overestimation of model accuracy and exaggeration of actual performance (40). We chose the more robust logistic regression model in our study. Our model did not perform worse than machine learning algorithms.

The HELENICC score is an excellent model for predicting mortality in patients with sepsis-related AKI undergoing CRRT (41), but not all patients with AKI undergoing CRRT have sepsis, and we hope that our model will be useful for clinical decision making in a larger number of patients with AKI.

The greatest advantage of this study over previous studies is that the external validation was based on completely independent data, and good model performance was achieved. This finding demonstrates the good generalizability of our model.

### 4.2. Implications for clinical practice

As statistician Professor Efron stated, in the absence of genius-level insight, statistical estimation theory is intended as an instrument for peering through noisy data and discerning a smooth underlying truth (42). Our models are not solely designed to predict patient outcomes but to offer new possibilities for clinicians and patient families to participate in shared decision-making regarding patient care.

We were able to quickly assess the risk of patient death with the nomogram and web-based prognostic calculator in this study, but some challenges exist.

On the one hand, although ICU physicians readily accept data-driven advice in their interactions with smart devices and the Internet, they remain cautious regarding the advice such technology provides in clinical decision-making (43). Even when models conclude that some AKI patients will not be able to reverse their deterioration even with CRRT, ICU physicians still prefer to treat them to the fullest. Physicians are always concerned that they are doing too little, and sometimes they are willing to do more than resuscitation interventions knowing that a treatment does not fundamentally change the patient's outcome (44). Using such technologies in clinical work must provide actionable information for the right patient at the right time. For example, outcomes can be predictive information to help clinicians make clinical decisions with some basis of reference. In addition, many factors that influence clinical decisions, including clinical, social, and personal factors, are not necessarily reflected in the digital record, thus any predictive results need to be evaluated, interpreted, and fleshed out by the clinician before any action is taken. Therefore, it is still the clinician who makes the final decision. Of course, this also requires critical care physicians to have some ability to interpret and use these results (43).

On the other hand, no medical practice is immune to ethical considerations, and the application of these technologies to the management of critically ill patients is fundamentally a medical practice for patients. This also requires compliance with medical ethical requirements.

It is important to emphasize that the inappropriate use of these technologies can cause harm to patients (45). Therefore, we must be cautious and ensure that it can be reasonably and safely tested and used in critically ill patients (46).

### 4.3. Weaknesses of the study

There are potential limitations in our study.

First, missing data are unavoidable in retrospective studies. Rather than excluding all patients with missing data from the analysis, we used multiple imputation to reduce the impact of missing data. With theoretical and empirical evidence of the technique's superiority to traditional complete case analysis, multiple interpolation has become widely accepted and is increasingly used (47, 48).

Second, because our development cohort was derived from the MIMIC-IV database, variables with significant predictive value that are easily accessible, such as the mean platelet volume and some widely reported biomarkers, were not included in our study. Han et al. (25) showed that the mean platelet volume may be an inexpensive and useful predictor of the 28-day all-cause mortality in AKI patients requiring CRRT. The predictive value of biomarkers such as tissue inhibitor metalloproteinase-2 (TIMP-2), insulin-like growth factor-binding protein 7 (IGFBP7) and neutrophil gelatinase-associated lipocalin (NGAL) has also been widely reported (49, 50). Unfortunately, these variables were not available in the MIMIC-IV database. These variables may need to be considered in future model updates.

Finally, our model seems to underestimate the mortality rate of patients. However, the performance during model development, internal validation, and external validation was in the acceptable range. Importantly, our validation cohort was completely independent of the development cohort in both time and space.

## 5. Conclusion

The prediction model we developed based on data from 1,148 patients from the MIMIC IV database reliably estimated outcomes in a fully independent validation cohort containing data from 121 patients. The predictor items are readily available, and the nomogram and the web-based prognostic calculator offer new possibilities for shared clinical decision-making between clinicians and patient families.

## Data availability statement

The original contributions presented in the study are included in the article/Supplementary material, further inquiries can be directed to the corresponding author/s.

## Ethics statement

The studies involving human participants were reviewed and approved by the Ethics Committee of the Fourth Hospital of Hebei Medical University. Written informed consent for participation was not required for this study in accordance with the national legislation and the institutional requirements.

## Author contributions

Study design: BL, YH, KZ, and ZH. Data collection: BL, LC, HZ, XW, and LL. Data analysis and drafting of the manuscript: BL. Data interpretation: BL, YH, and KZ. Revising the manuscript content: YH and KZ. Approving the final version of the manuscript: ZH. All authors contributed to the article and approved the submitted version.

## Conflict of interest

The authors declare that the research was conducted in the absence of any commercial or financial relationships that could be construed as a potential conflict of interest.

## Publisher's note

All claims expressed in this article are solely those of the authors and do not necessarily represent those of their affiliated organizations, or those of the publisher, the editors and the reviewers. Any product that may be evaluated in this article, or claim that may be made by its manufacturer, is not guaranteed or endorsed by the publisher.

## References

[1] Hoste EAJ Kellum JA Selby NM Zarbock A Palevsky PM Bagshaw SM. Global epidemiology and outcomes of acute kidney injury. Nat Rev Nephrol. (2018) 14:607–25. 10.1038/s41581-018-0052-0 30135570

[2] Negi S Koreeda D Kobayashi S Yano T Tatsuta K Mima T. Acute kidney injury: epidemiology, outcomes, complications, and therapeutic strategies. Semin Dial. (2018) 31:519–27. 10.1111/sdi.12705 29738093

[3] Ronco C Bellomo R Kellum JA. Acute kidney injury. Lancet. (2019) 394:1949–64. 10.1016/S0140-6736(19)32563-2 31777389

[4] Hoste EAJ Bagshaw SM Bellomo R Cely CM Colman R Cruz DN. Epidemiology of acute kidney injury in critically ill patients: the multinational AKI-EPI study. Intens Care Med. (2015) 41:1411–23. 10.1007/s00134-015-3934-7 26162677

[5] Rabindranath K Adams J Macleod AM Muirhead N. Intermittent versus continuous renal replacement therapy for acute renal failure in adults. Cochrane Database Syst Rev. (2007) CD003773. 10.1002/14651858.CD003773.pub3 17636735 PMC13199906

[6] Schneider AG Bellomo R Bagshaw SM Glassford NJ Lo S Jun M. Choice of renal replacement therapy modality and dialysis dependence after acute kidney injury: a systematic review and meta-analysis. Intens Care Med. (2013) 39:987–97. 10.1007/s00134-013-2864-5 23443311

[7] Nash DM Przech S Wald R O'Reilly D. Systematic review and meta-analysis of renal replacement therapy modalities for acute kidney injury in the intensive care unit. J Crit Care. (2017) 41:138–44. 10.1016/j.jcrc.2017.05.002 28525779

[8] Uchino S Bellomo R Morimatsu H Morgera S Schetz M Tan I. Continuous renal replacement therapy: a worldwide practice survey. The beginning and ending supportive therapy for the kidney (B.E.S.T. Kidney) investigators. Intensive Care Med. (2007) 33:1563–70. 10.1007/s00134-007-0754-4 17594074

[9] Steyerberg EW Moons KGM van der Windt DA Hayden JA Perel P Schroter S. Prognosis research strategy (PROGRESS) 3: prognostic model research. PLoS Med. (2013) 10:e1001381. 10.1371/journal.pmed.1001381 23393430 PMC3564751

[10] Knaus WA Draper EA Wagner DP Zimmerman JE. APACHE II: a severity of disease classification system. Crit Care Med. (1985) 13:818–29. 10.1097/00003246-198510000-00009 3928249

[11] Vincent JL Moreno R Takala J Willatts S De Mendonça A Bruining H. The SOFA (sepsis-related organ failure assessment) score to describe organ dysfunction/failure. On behalf of the working group on sepsis-related problems of the european society of intensive care medicine. Intens Care Med. (1996) 22:707–10. 10.1007/BF01709751 8844239

[12] Kim Y Park N Kim J Kim DK Chin HJ Na KY. Development of a new mortality scoring system for acute kidney injury with continuous renal replacement therapy. Nephrology. (2019) 24:1233–40. 10.1111/nep.13661 31487094

[13] Kang MW Kim J Kim DK Oh KH Joo KW Kim YS. Machine learning algorithm to predict mortality in patients undergoing continuous renal replacement therapy. Crit Care. (2020) 24:42. 10.1186/s13054-020-2752-7 32028984 PMC7006166

[14] Johnson A Bulgarelli L Pollard T Horng S Celi LA Mark R. MIMIC-IV (Version 1.0). PhysioNet. (2021). 10.13026/s6n6-xd98

[15] Goldberger AL Amaral LA Glass L Hausdorff JM Ivanov PC Mark RG. PhysioBank, PhysioToolkit, and PhysioNet: components of a new research resource for complex physiologic signals. Circulation. (2000) 101:E215–20. 10.1161/01.CIR.101.23.e215 10851218

[16] Levin A Stevens PE Bilous RW Coresh J Francisco ALMD Jong PED. Kidney disease: improving global outcomes (KDIGO) CKD work group. KDIGO 2012 clinical practice guideline for the evaluation and management of chronic kidney disease. Kidney Int Suppl. (2013) 3:1–150. 10.1038/kisup.2012.7323732715

[17] Lines SW Cherukuri A Murdoch SD Bellamy MC Lewington AJP. The outcomes of critically ill patients with acute kidney injury receiving renal replacement therapy. Int J Artif Organs. (2011) 34:2–9. 10.5301/IJAO.2011.6312 21308666

[18] Demirjian S Chertow GM Zhang JH O'Connor TZ Vitale J Paganini EP. Model to predict mortality in critically ill adults with acute kidney injury. clinical journal of the american society of nephrology: CJASN. (2011) 6:2114–20. 10.2215/CJN.02900311 21896828 PMC3359007

[19] Stads S Fortrie G van Bommel J Zietse R Betjes MGH. Impaired kidney function at hospital discharge and long-term renal and overall survival in patients who received CRRT. Clin J Am Soc Nephrol. (2013) 8:1284–91. 10.2215/CJN.06650712 23599403 PMC3731903

[20] De Corte W Dhondt A Vanholder R De Waele J Decruyenaere J Sergoyne V. Long-term outcome in ICU patients with acute kidney injury treated with renal replacement therapy: a prospective cohort study. Crit Care. (2016) 20:256. 10.1186/s13054-016-1409-z 27520553 PMC4983760

[21] Katayama S Uchino S Uji M Ohnuma T Namba Y Kawarazaki H. Factors predicting successful discontinuation of continuous renal replacement therapy. Anaesth Intens Care. (2016) 44:453–7. 10.1177/0310057X1604400401 27456174

[22] Oh HJ Park JT Kim JK Yoo DE Kim SJ Han SH. Red blood cell distribution width is an independent predictor of mortality in acute kidney injury patients treated with continuous renal replacement therapy. Nephrol Dial Transpl. (2012) 27:589–94. 10.1093/ndt/gfr307 21712489

[23] Jung SY Kim H Park S Jhee JH Yun HR Kim H. Electrolyte and mineral disturbances in septic acute kidney injury patients undergoing continuous renal replacement therapy. Medicine. (2016) 95:e4542. 10.1097/MD.0000000000004542 27603344 PMC5023866

[24] Jung SY Kwon J Park S Jhee JH Yun HR Kim H. phosphate is a potential biomarker of disease severity and predicts adverse outcomes in acute kidney injury patients undergoing continuous renal replacement therapy. PLoS ONE. (2018) 13:e0191290. 10.1371/journal.pone.0191290 29415048 PMC5802883

[25] Han JS Park KS Lee MJ Kim CH Koo HM Doh FM. Mean platelet volume is a prognostic factor in patients with acute kidney injury requiring continuous renal replacement therapy. J Crit Care. (2014) 29:1016–21. 10.1016/j.jcrc.2014.07.022 25138689

[26] Vickers AJ Elkin EB. Decision curve analysis: a novel method for evaluating prediction models. Med Decis Making. (2006) 26:565–74. 10.1177/0272989X06295361 17099194 PMC2577036

[27] Kerr KF Brown MD Zhu K Janes H. Assessing the clinical impact of risk prediction models with decision curves: guidance for correct interpretation and appropriate use. J Clin Oncol. (2016) 34:2534–40. 10.1200/JCO.2015.65.5654 27247223 PMC4962736

[28] Meersch M Schmidt C Hoffmeier A Van Aken H Wempe C Gerss J. Prevention of cardiac surgery-associated AKI by implementing the KDIGO guidelines in high risk patients identified by biomarkers: the PrevAKI randomized controlled trial. Intens Care Med. (2017) 43:1551–61. 10.1007/s00134-016-4670-3 28110412 PMC5633630

[29] Göcze I Jauch D Götz M Kennedy P Jung B Zeman F. Biomarker-guided intervention to prevent acute kidney injury after major surgery: the prospective randomized BigpAK study. Ann Surg. (2018) 267:1013–20. 10.1097/SLA.0000000000002485 28857811

[30] Selby NM Casula A Lamming L Stoves J Samarasinghe Y Lewington AJ. An organizational-level program of intervention for AKI: a pragmatic stepped wedge cluster randomized trial. J Am Soc Nephrol. (2019) 30:505–15. 10.1681/ASN.2018090886 31058607 PMC6405151

[31] Sanchez-Pinto LN Luo Y Churpek MM. Big data and data science in critical care. Chest. (2018) 154:1239–48. 10.1016/j.chest.2018.04.037 29752973 PMC6224705

[32] Sutherland SM Goldstein SL Bagshaw SM. Acute kidney injury and big data. Contrib Nephrol. (2018) 193:55–67. 10.1159/000484963 29393191

[33] Sutherland SM Chawla LS Kane-Gill SL Hsu RK Kramer AA Goldstein SL. Utilizing electronic health records to predict acute kidney injury risk and outcomes: workgroup statements from the 15(Th) ADQI consensus conference. Can J Kidney Health Dis. (2016) 3:11. 10.1186/s40697-016-0099-4 26925247 PMC4768420

[34] Hodgson LE Sarnowski A Roderick PJ Dimitrov BD Venn RM Forni LG. Systematic review of prognostic prediction models for acute kidney injury (AKI) in general hospital populations. BMJ Open. (2017) 7:e016591. 10.1136/bmjopen-2017-016591 28963291 PMC5623486

[35] Koyner JL Adhikari R Edelson DP Churpek MM. Development of a multicenter ward-based AKI prediction model. Clin J Am Soc Nephrol. (2016) 11:1935–43. 10.2215/CJN.00280116 27633727 PMC5108182

[36] Malhotra R Kashani KB Macedo E Kim J Bouchard J Wynn S. A risk prediction score for acute kidney injury in the intensive care unit. Nephrol Dial Transpl. (2017) 32:814–22. 10.1093/ndt/gfx026 28402551

[37] Bhatraju PK Zelnick LR Katz R Mikacenic C Kosamo S Hahn WO. A prediction model for severe aki in critically ill adults that incorporates clinical and biomarker data. Clin J Am Soc Nephrol. (2019) 14:506–14. 10.2215/CJN.04100318 30917991 PMC6450340

[38] Leaf DE Waikar SS. End points for clinical trials in acute kidney injury. Am J Kidney Dis. (2017) 69:108–16. 10.1053/j.ajkd.2016.05.033 27599630 PMC5182076

[39] Sukmark T Lumlertgul N Praditpornsilpa K Tungsanga K Eiam-Ong S Srisawat N. SEA-MAKE score as a tool for predicting major adverse kidney events in critically ill patients with acute kidney injury: results from the SEA-AKI study. Ann Intens Care. (2020) 10:42. 10.1186/s13613-020-00657-9 32300902 PMC7162998

[40] Obermeyer Z Emanuel EJ. Predicting the future - big data, machine learning, and clinical medicine. N Engl J Med. (2016) 375:1216–9. 10.1056/NEJMp1606181 27682033 PMC5070532

[41] da Hora Passos R Ramos JGR Mendonça EJB Miranda EA Dutra FRD Coelho MFR. A clinical score to predict mortality in septic acute kidney injury patients requiring continuous renal replacement therapy: the HELENICC score. BMC Anesthesiol. (2017) 17:21. 10.1186/s12871-017-0312-8 28173756 PMC5297177

[42] Efron B. Prediction, estimation, and attribution. J Am Stat Assoc. (2020) 115:636–55. 10.1080/01621459.2020.1762613

[43] Verghese A Shah NH Harrington RA. What this computer needs is a physician: humanism and artificial intelligence. JAMA. (2018) 319:19–20. 10.1001/jama.2017.19198 29261830

[44] Gawande A. Being Mortal: Illness, Medicine, and What Matters in the End. London: Profile Books (2014). p. 282.

[45] Han YY Carcillo JA Venkataraman ST Clark RSB Watson RS Nguyen TC. Unexpected increased mortality after implementation of a commercially sold computerized physician order entry system. Pediatrics. (2005) 116:1506–12. 10.1542/peds.2005-1287 16322178

[46] Ghassemi M Celi LA Stone DJ. State of the art review: the data revolution in critical care. Crit Care. (2015) 19:118. 10.1186/s13054-015-0801-4 25886756 PMC4361206

[47] Bounthavong M Watanabe JH Sullivan KM. Approach to addressing missing data for electronic medical records and pharmacy claims data research. Pharmacotherapy. (2015) 35:380–7. 10.1002/phar.1569 25884526

[48] Austin PC White IR Lee DS van Buuren S. Missing data in clinical research: a tutorial on multiple imputation. Can J Cardiol. (2020) 37:1322–31. 10.1016/j.cjca.2020.11.010 33276049 PMC8499698

[49] Xie Y Ankawi G Yang B Garzotto F Passannante A Breglia A. Tissue inhibitor metalloproteinase-2 (TIMP-2) IGF-binding protein-7 (IGFBP7) levels are associated with adverse outcomes in patients in the intensive care unit with acute kidney injury. Kidney Int. (2019) 95:1486–93. 10.1016/j.kint.2019.01.020 30982674

[50] Kümpers P Hafer C Lukasz A Lichtinghagen R Brand K Fliser D. Serum neutrophil gelatinase-associated lipocalin at inception of renal replacement therapy predicts survival in critically ill patients with acute kidney injury. Crit Care. (2010) 14:R9. 10.1186/cc8861 20122150 PMC2875521

